# Maternity in adolescence in Brazil: high fertility rates and stark inequalities across municipalities and regions

**DOI:** 10.1590/0102-311XEN057625

**Published:** 2026-02-06

**Authors:** Aluísio J. D. Barros, Cauane Blumenberg, Janaína Calu Costa, Luis Paulo Vidaletti Ruas, Natália P. Lima, Fernando César Wehrmeister, Francine dos Santos Costa, Luiza Eunice Sá da Silva, Cesar G. Victora

**Affiliations:** 1 Centro Internacional de Equidade em Saúde, Universidade Federal de Pelotas, Pelotas, Brasil.; 2 Faculdade de Saúde Pública, Universidade de São Paulo, São Paulo, Brasil.

**Keywords:** Live Birth, Adolescent Mothers, Fecundity Rate, Health Status Disparities, Nacimiento Vivo, Madres Adolescente, Índice de Fecundidad, Desigualdad en Salud

## Abstract

Adolescent childbearing is a major public health challenge in Brazil, with a fertility rate of 43.6 births per 1,000 girls aged 15-19 years in 2022. This study investigated inequalities in adolescent fertility rates across Brazilian municipalities and regions and explored their association with sociodemographic characteristics, namely municipal deprivation and population size. Data from the Brazilian Information System on Live Births and the 2022 *Demographic Census* were analyzed. The study included births occurring between 2020 and 2022, excluding municipalities with fewer than 50 births over the three-year period. Municipal adolescent fertility rates were compared with average estimates observed in countries classified by income level and described according to quintiles of the Brazilian Deprivation Index and population size. Inequalities within each geographic region were assessed using the mean absolute difference from the regional mean and range. Substantial disparities in adolescent fertility rates were found across regions, with the North and Northeast exhibiting the highest estimates, while the South and Southeast showed comparatively lower rates. The Central-West presented intermediate values. We also found vast inequalities across municipalities, with a small proportion − mostly in the North − experiencing exceptionally high adolescent fertility rates. Municipalities with higher levels of deprivation had markedly higher adolescent fertility rates, underscoring the influence of broader socioeconomic factors on adolescent fertility. These results emphasize the need for targeted interventions and policies that address these underlying contextual determinants to effectively reduce fertility rates among girls in Brazil.

## Abstract (es)

La maternidad adolescente es un importante problema de salud pública en Brasil, con una tasa de fertilidad de 43,6 nacimientos por cada mil niñas de entre15 y 19 años en 2022. Este estudio investigó las desigualdades en las tasas de fertilidad adolescente en los municipios y regiones brasileños y exploró la asociación entre la tasas de fertilidad adolescente y las características sociodemográficas, la privación municipal y el tamaño de la población. Se utilizaron datos del Sistema Nacional de Información sobre Nacidos Vivos y del *Censo Demográfico* de 2022. Los análisis incluyeron los nacimientos entre 2020 y 2022, excluyendo los municipios con menos de 50 nacimientos durante el período de tres años. Las tasas de mortalidad infantil municipal se compararon con la estimación media observada en los países clasificados por nivel de ingresos y se describieron según los quintiles del Índice de Privación de Brasil y el tamaño de la población. Las desigualdades dentro de cada región geográfica se evaluaron utilizando la diferencia absoluta media con respecto a la media y el rango regionales. Se observaron disparidades sustanciales en la tasa de mortalidad infantil entre las regiones, con el Norte y el Noreste presentando las estimaciones más altas, mientras que el Sur y el Sureste mostraron tasas comparativamentemás bajas; el Centro0Oeste presentó valores intermedios. Encontramos grandes desigualdades entre los municipios, y una pequeña proporción de ellos, concentrados en la Región Norte, experimentaron una tasas de fertilidad adolescente excepcionalmente alta. Los municipios con mayores niveles de privación presentaban una tasas de fertilidad adolescente notablemente más elevada, lo que pone de relieve la influencia de factores socioeconómicos más amplios en la fecundidad adolescente. Los resultados subrayan la necesidad de intervenciones y políticas específicas que aborden estos determinantes contextuales fundamentales para reducir de manera eficaz las tasas de fecundidad entre las niñas en Brasil.

## Introduction

Adolescent childbearing remains a major public health challenge in Brazil. According to World Bank estimates (dataset v2_898), Brazil’s adolescent fertility rate in 2022 was 43.6 births per 1,000 girls aged 15-19, surpassing its BRICS counterparts − namely Russia, India, and China − which had a maximum adolescent fertility rate of 14.2 ^1^. Brazil’s rate is also higher than the average of upper-middle-income countries, which stands at 27.8. Although fertility and adolescent motherhood have declined in recent years, this reduction has been uneven across sociodemographic groups ^2^ ^,^ ^3^ ^,^ ^4^.

Brazil’s high adolescent fertility rate contrasts with its low total fertility rate − 1.6 in 2023, below the replacement level − and the high accessibility of contraception, as indicated by the large proportion of women of reproductive age using modern contraceptives (84% in 2019) ^5^ ^,^ ^6^. This suggests that family planning programs may not be adequately reaching adolescents, or that those living in unfavorable contexts, with limited life opportunities and restricted access to education or health services, are more likely to experience early pregnancy ^7^.

Having a child during adolescence has numerous adverse consequences for health, education, and economic stability. Health risks include higher rates of complications such as anemia, preterm labor, and low birth weight due to the physiological immaturity of young mothers ^8^ ^,^ ^9^ ^,^ ^10^. Educationally, teenage mothers often face significant setbacks, including higher dropout rates and limited academic achievement, which in turn restrict future opportunities ^8^ ^,^ ^9^ ^,^ ^10^ ^,^ ^11^ ^,^ ^12^ ^,^ ^13^ ^,^ ^14^. Economically, young mothers are more likely to experience financial instability and poverty, as disrupted education and parenting responsibilities make securing stable employment more difficult ^15^ ^,^ ^16^. Thus, adolescent childbearing is an undesirable outcome for girls, their families, and society, resulting in lower levels of social development. It is essential to note that the risks associated with pregnancy differ between younger adolescents (less than 14) and older adolescents (15-19); the former group, according to Brazilian law, involves statutory rape (Article 217-A of the *Brazilian Penal Code* ^17^). Therefore, despite their smaller numbers, adolescents under 14 should not be overlooked when studying adolescent childbearing.

Although several individual-level factors may influence adolescent fertility, such as educational attainment ^18^ and intergenerational effects ^19^, subnational inequalities and community and contextual characteristics have been highlighted as important determinants ^7^ ^,^ ^20^ ^,^ ^21^.

Therefore, identifying who adolescent mothers are, where they live, and what drives early childbearing is essential to understand the phenomenon and establish policies to decrease adolescent fertility rates.

This study investigated inequalities in adolescent fertility rate across Brazilian municipalities using data from the Brazilian Information System on Live Birth (SINASC, acronym in Portuguese). It aimed to highlight disparities across municipalities and regions, identify areas with the highest occurrence, and assess the association between social vulnerability and early childbearing.

## Methods

This study utilized data from SINASC ^22^ for 2020-2022, obtained from OpenDataSUS ^23^. Population statistics by sex and age were also obtained from the 2022 Brazilian Census, provided by the Brazilian Institute of Geography and Statistics (IBGE, acronym in Portuguese) ^24^.

Adolescent fertility rate were calculated by dividing the number of births to adolescents aged 10-14 and 15-19 between 2020 and 2022 in each municipality by three times the corresponding population estimates from the 2022 *Brazilian Demographic Census*, aiming to annualize the rates. Municipalities with fewer than 50 births over the three-year period were excluded from the analysis. Using data from three years improved the stability of estimates and reduced possible noise, as births to girls under 20 years account for only about 13% of total births. When estimating median and mean Adolescents fertility rates by groups of municipalities, rates were weighted by the size of the corresponding population.

To enable comparison and account for the absence of standard classification thresholds, adolescent fertility rate bands for the 15-19 age group were created using the average adolescent fertility rate estimates observed in groups of countries classified by income level according to the World Bank, using 2022 data: high-income (11.2 births per 1,000 adolescent girls), upper-middle-income (27.8), lower-middle-income (44.7), and low-income (94.0) (API_SP.ADO.TFRT_DS2_en_excel_v2_898.xlsx) ^1^. The approximate midpoints between these values were used as cut-offs to create fertility rate bands: < 20, 20 |-- 40, 40 |-- 65, and 65+, corresponding to high-, upper-middle-, lower-middle-, and low-income countries, respectively. Based on these bands, the number and proportion of Brazilian municipalities falling within each category were calculated.

Municipal inequality within Brazil’s geographic regions (North, Northeast, Southeast, South, and Central-West) was assessed using two indicators: the mean absolute difference to the regional mean (MADM) and the range. MADM is defined as $\frac{\sum \left|y_{i}-\bar{y}\right|}{N}$ , in which *y*_*i*_ is the municipal adolescent fertility rate, ӯ is the regional mean, and N is the number of municipalities within each region. The range was calculated as the difference between the maximum and minimum adolescent fertility rate values within each region.

The Brazilian Deprivation Index (IBP, acronym in Portuguese) ^25^ was used to assess municipal deprivation levels. This multidimensional indicator combines data on (i) the percentage of households earning less than half the Brazilian minimum wage, (ii) the percentage of individuals aged seven and older who are illiterate, and (iii) the percentage of the population without adequate access to sewage, clean water, and waste collection, as well as bathroom or shower facilities. Unlike the Human Development Index (HDI), the IBP does not include child mortality or life expectancy, as these indicators may be affected by the level of adolescent fertility rate. The most recent version of the IBP is based on data from the 2010 Brazilian Census. Municipalities were ranked according to their IBP values and divided into five equally sized groups, unweighted by population, to ensure an equal number of units in each group rather than an equal population count. Furthermore, population size (categorized as < 5, 5-, 10-, 50-, and 100+ thousand inhabitants) was analyzed as a potential predictor of adolescent fertility rate, providing additional context to the observed fertility patterns.

The estimation of adolescent fertility rate by IBP quintiles and municipal population size across geographic regions employed linear regression models, including interaction terms between the predictors and region, which were tested for significance. All statistical analyses and figures were produced using Stata (https://www.stata.com).

The analyses utilized anonymized data from publicly available sources and therefore do not require ethical approval.

## Results

A total of 7,968,916 birth records from 2020-2022 were retrieved from SINASC. Of these, 49,325 (0.62%) were births to girls aged 10-14 and 1,012,640 (12.7%) to adolescents aged 15-19. Age information was missing in only 141 records. After excluding 68 municipalities with fewer than 50 births to adolescents aged 10-19, the final analytical sample comprised 5,502 municipalities (98.8%).

**Figure 1:**
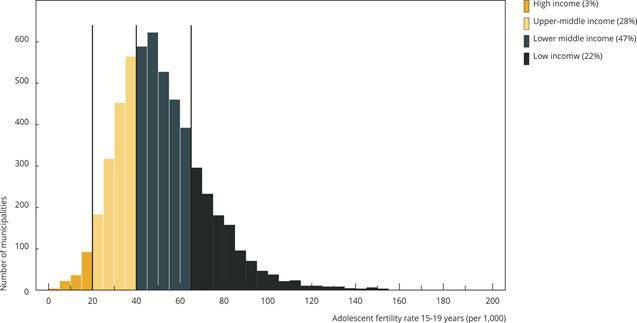
Distribution of adolescent fertility rates (15-19 years) across 5,502 Brazilian municipalities, 2020-2022.

The median municipal adolescent fertility rate for girls aged 10-14 was 1.9 births per 1,000, while the median for those aged 15-19 was substantially higher, at 43.3 births per 1,000, ranging from zero to 201. The adolescent fertility rate distribution for the 15-19 age group resembled a right-skewed Gaussian distribution (Figure 1), indicating a small but relevant proportion of municipalities with very high adolescents fertility rates. Figure 1 also indicates that 22% of municipalities fell within the low-income adolescent fertility rate band (estimates above 65 births per 1,000), while 47% fell within the lower-middle income band. Only 28% fell within Brazil’s own upper-middle-income band, and 3% in the high-income band.

Nota: the histogram is divided into bands according to the mean adolescent fertility rate values of high-, upper-middle-, lower-middle-, and low-income countries. Percentages indicate the proportion of municipalities within each band.

**Figure 2:**
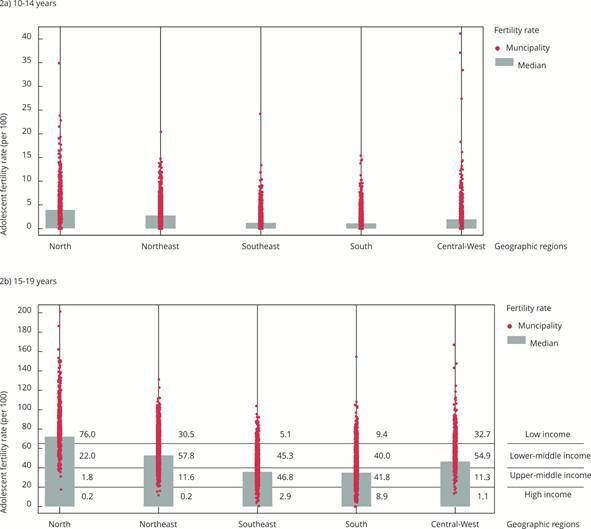
Adolescent fertility rates across 5,502 Brazilian municipalities by geographic region, with the median rate for each region. Brazil, 2020-2022.

Note: 2a) shows rates for adolescents aged 10-14 years, and 2b) for adolescents aged 15-19 years. 2b) also indicates the percentages of municipalities in each fertility rate band, defined by the mean rates of high, upper-middle, lower-middle, and low-income countries.

Substantial regional disparities in adolescents fertility rates were evident. For young adolescents aged 10-14, the North exhibited a median adolescent fertility rate nearly four times higher than that of the South. The regional median adolescent fertility rate estimates (births per 1,000) were, in decreasing order: 4% (North), 2.8% (Northeast), 2% (Central-West), 1.2% (Southeast), and 1.1% (South). Ten municipalities nationwide recorded adolescents fertility rates of 20% or more for this younger age group (Figure 2a).

Among adolescents aged 15-19, the North median adolescent fertility rate (77.1%) was more than twice that of the South (35%) (Table 1 and Figure 2b). Within all five regions, adolescents fertility ratess varied widely at the municipal level, with both low and high values observed in every region. Notably, 148 municipalities recorded adolescent fertility rate of 100 or more among girls 15-19 years.

**Table 1:** Median adolescent fertility rate (15-19 years) by geographic region and nationally, and number and proportion of municipalities with adolescent fertility rates within bands defined by the mean rate of high-, upper-middle-, lower-middle-, and low-income countries. Brazil, 2020-2022.

Geographic region	All municipalities		Adolescent fertility rate bands (births/1,000)			
	N	Median (adolescent fertility rate per 1,000)	< 20 high-income band	20 |-- 40 upper-middle-income band	40 |-- 65 lower-middle-income band	65+ lower-income band
			% municipalities	% municipalities	% municipalities	% municipalities
North	450	77.1	0.2	1.8	22.0	76.0
Northeast	1,793	52.8	0.2	11.6	57.7	30.5
Southeast	1,638	35.8	2.9	46.7	45.3	5.1
South	1,162	35.0	8.8	41.8	40.0	9.4
Central-West	459	46.6	1.1	11.3	54.9	32.7
Total	5,502	43.3	2.9	27.6	47.1	22.4

Note: the table includes 5,502 municipalities with 50 or more births to adolescents aged 10-19 from 2020 to 2022.

Figure 2b also presents the percentage of municipalities in each income group’s adolescent fertility rate band for adolescents aged 15-19, by region. The North showed the highest percentage of municipalities (76%) in the low-income adolescent fertility rate band, contrasting with the Southeast and South, which exhibited more favorable distributions although roughly half of their municipalities still fell within the lower-middle and low-income bands. None of the regions had most of the municipalities within Brazil’s upper-middle income band.

Measures of municipal inequalities within regions, as seen in the figures, are presented in Table 2. For girls aged 10-14, the North and Central-West showed the highest inequalities, with MADM values markedly higher compared to other regions. Among adolescents aged 15-19, the North and Central-West also presented the highest MADM values, although differences were less pronounced compared to the Northeast and South. The measures confirm the visual impression of high inequalities in all five regions. The ranges, reflecting the difference between highest to lowest adolescents fertility rates, exceeded 100 births per 1,000 in three regions, representing a huge gap.

**Table 2:** Mean absolute difference to the mean (MADM) and range of adolescent fertility rates by geographic region. Brazil, 2020-2022.

Geographic region	Aged 10-14 years		Aged 15-19 years	
	MADM	Range	MADM	Range
North	3.1	28.9	20.5	117.6
Northeast	1.7	17.1	13.0	73.8
Southeast	1.3	22.7	10.3	63.6
South	1.6	13.5	13.4	114.0
Central-West	2.3	37.8	14.5	107.5

Note range − difference between the maximum and minimum values within the region.

**Figure 3:**
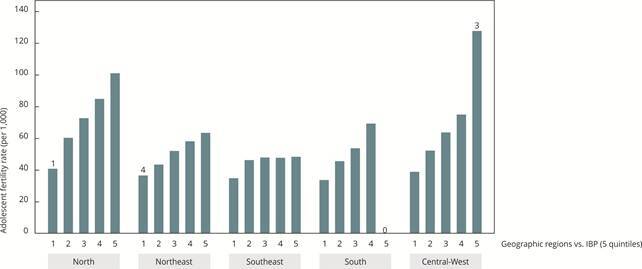
Mean adolescent fertility rates (15-19 years) across municipalities by quintile of the Brazilian Deprivation Index (IBP, acronym in Portuguese) by geographic region. Brazil, 2020-2022.

Note: numbers displayed above some bars indicate the number of municipalities in each group when less than 10. Deprivation quintiles: 1 = least deprived up to 5 = most deprived.

Municipal deprivation, as measured by the IBP, was strongly associated with adolescent fertility rate (Figure 3 and Tables 3 and 4). The regression model including IBP, geographic region, and their interaction accounted for 64.6% of the adolescent fertility rate variability in the 15-19 age group. Except for the North, adolescent fertility rate levels within the same deprivation quintile were relatively similar across regions, with a consistent gradient of adolescent fertility rate as deprivation increased. In the North, adolescents fertility rates were systematically higher across all IBP quintiles compared to other regions, except for the least deprived group, which had only one municipality. Notably, no municipalities in the South were in the most deprived group. The Central-West presented a much higher adolescent fertility rate in the most deprived group; however, this estimate was based on only three municipalities.

**Table 3:** Adolescent fertility rates (births per 1,000 adolescent girls aged 15-19) by geographic region and Brazilian Deprivation Index (IBP, acronym in Portuguese) quintiles.

Geographic region	IBP (quintiles)				
	Q1 (least deprived)	Q2	Q3	Q4	Q5 (most deprived)
North	40.9	60.4	72.8	85.0	101.1
Northeast	36.6	43.5	52.1	58.2	63.5
Southeast	34.9	46.4	48.0	47.8	48.4
South	33.8	45.7	53.8	69.4	N/A
Central-West	39.0	52.3	63.8	75.1	127.8

Note: estimates derived from a linear regression model including geographic region, IBP quintiles, and their interaction (all p-values < 0.001). The R^2^ for the model was 0.646.

**Table 4:** Number of municipalities in each Brazilian Deprivation Index (IBP, acronym in Portuguese) quintile and geographic region.

Geographic region	IBP (quintiles)				
	Q1 (least deprived)	Q2	Q3	Q4	Q5 (most deprived)
North	1	19	104	193	132
Northeast	4	21	127	686	955
Southeast	646	491	312	167	22
South	411	416	305	27	0
Central-West	26	136	253	40	3

Note: five municipalities did not have an IBP estimate.

**Figure 4:**
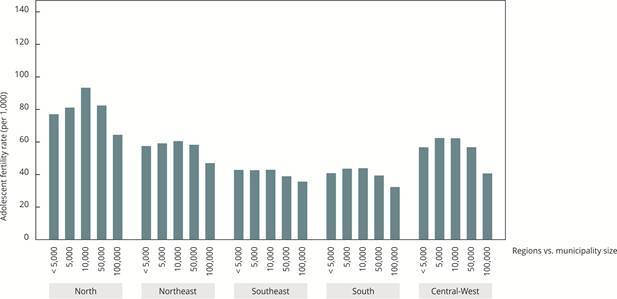
Mean adolescent fertility rates (15-19 years) across municipalities in each population size group by geographic region. Brazil, 2020-2022.

Municipality size was significantly associated with adolescent fertility rate among girls aged 15-19, although the effect size was much smaller than that of deprivation. Overall, we observed an inverted U-shaped association with maximum adolescent fertility rate occurring in municipalities with populations between 10,000 and 50,000 inhabitants. These results are presented in Figure 4 and Tables 5 and 6. We included population size in a model already containing IBP to assess the additional explanatory contribution of municipal size beyond deprivation. While population size remained significant when adjusted for deprivation, the model’s R^2^ increased by only 0.4 percentage points (data not shown).

**Table 5:** Adolescent fertility rates (births per 1,000 adolescent girls aged 15-19) by geographic region and municipality size.

Geographic region	Municipality size (total population)				
	< 5,000	5,000	10,000	50,000	100,000
North	77.1	81.1	93.4	82.4	64.4
Northeast	57.5	59.2	60.6	58.3	47.0
Southeast	42.9	42.7	42.9	38.9	35.6
South	40.9	43.6	43.9	39.4	32.4
Central-West	56.8	62.4	62.3	56.8	40.7

Note: estimates derived from a linear regression model including geographic region, groups of municipality size, and their interaction (all p-values < 0.001). The R^2^ for the model was 0.564.

**Table 6:** Number of municipalities in each population size group and geographic region.

Geographic region	Municipality size (total population)				
	0	5,000	10,000	50,000	100,000
North	92	66	221	45	26
Northeast	247	372	999	111	64
Southeast	367	374	640	108	149
South	412	262	378	55	55
Central-West	138	96	181	19	25

## Discussion

This study reveals significant inequalities in adolescent fertility rate across more than 5,000 Brazilian municipalities from 2020-2022. Substantial disparities were observed both nationally and within geographical regions, which have more homogeneous populations.

A comparison of adolescents fertility rates among adolescents aged 15-19 with those of countries of different income levels revealed that 70% of Brazilian municipalities presented rates above those of upper-middle-income countries, Brazil’s own income classification. The situation is critical particularly in the North, where 98% of municipalities surpass the upper-middle-income countries range. Furthermore, a large proportion of the municipal variability in adolescent fertility rate was explained by the level of municipal deprivation, providing an important indication as to what can be done to reduce the high rates observed.

The substantial regional and municipal variability observed in adolescent fertility rate, particularly among girls aged 15-19, reveals a complex and uneven landscape of adolescent childbearing in Brazil. The strong effect of municipal deprivation on adolescents fertility rates underscores the relevance of socioeconomic and human development factors, as well as the structural influence of social inequality, in shaping this outcome. Previous studies have reported associations between adolescent fertility rate and poverty, limited access to health services ^26^, and low education attainment ^27^. Our findings align with prior literature describing the heterogeneous distribution of births to adolescent girls across Brazil and their association with contextual indicators such as the municipal Human Development Index and income inequality, measured by the Gini coefficient ^20^ ^,^ ^28^. Addressing poverty, improving access to education − particularly for girls − and enhancing family planning services should be central to effective interventions. Additionally, the lack of long-term perspectives for an improved quality of life may hamper adolescents’ ability to plan for the future, leading some to view early childbearing as a means of gaining social status, security, or companionship ^29^.

The consistently higher adolescents fertility rates in the North, across all levels of deprivation, highlight the complex interplay of socioeconomic factors and other potential region-specific influences. These may include cultural norms, healthcare accessibility, and availability of family planning resources ^28^. Additional elements, such as a higher proportion of Indigenous populations, geographic remoteness, limited access to health services, and greater overall deprivation, warrant further investigation. Public policies targeting these areas should adopt cross-sectoral actions that integrate comprehensive sexual and reproductive education in schools, expand access to contraceptive methods, and strengthen primary healthcare systems with a focus on adolescent and women’s health. The presence of high adolescents fertility rates even in regions with better overall indicators, such as the Southeast and South, highlights the importance of identifying pockets of vulnerability within seemingly more advantaged settings. This heterogeneity suggests that uniform, nationwide interventions may be insufficient and reinforces the need for territorially tailored strategies responsive to specific regional and local contexts.

The number of births to adolescents aged 10-14 is particularly concerning, despite being a small proportion of births. We found 49,325 births among girls in this age group from 2020-2022 − an average of 16,441 births per year. According to Brazilian legislation, such pregnancies are considered the result of statutory rape, prompting immediate and vigorous actions to eliminate their occurrence with initiatives that protect girls and ensure their sexual and reproductive rights. When pregnancies among girls under 15 cannot be prevented, timely and unobstructed access to legal abortion, when chosen, must be guaranteed.

This study benefits from the high coverage of SINASC data, the near-universal rate of institutional deliveries across all social groups and municipalities, minimal missing values, national scope, and large sample size. However, as an ecological study relying on secondary data, it has limited capacity to explore individual-level determinants of adolescent fertility. Further research integrating mixed-methods approaches could deepen the understanding of these complex relationships. Our analyses focused exclusively on live births, thus excluding stillbirths, miscarriage, and abortions. Consequently, we do not present the full spectrum of adolescent pregnancies, although most cases are likely represented. The relatively small number of municipalities in the most deprived quintiles in certain regions limits the generalizability of findings for those contexts. It is important to note that we used IBP based on 2010 Census data, as no updated version is currently available. Therefore, changes in municipal deprivation since 2010 are not captured in our analysis. Nonetheless, the observed association was clear and strong.

It is also important to consider that the study period (2020-2022) coincided with the COVID-19 pandemic. This context may have influenced adolescent fertility patterns across Brazil, as the pandemic has disrupted access to health services − including contraceptive provision and sexual and reproductive healthcare − particularly in regions already facing structural weaknesses. School closures and reduced access to education and social protection programs may have further increased vulnerabilities among adolescents, especially in deprived areas. While our data cannot isolate specific effects of the pandemic, the observed disparities in adolescents fertility rates may partially reflect its uneven impact across regions and municipalities, potentially exacerbating pre-existing inequalities. This context reinforces the importance of resilient and equitable health and social systems capable of maintaining essential services during crises. Previous studies have also revealed that the effects of crises, including the COVID-19 pandemic, were smaller than initially speculated, given the pre-existing downward trend in adolescent fertility ^4^.

The findings highlight the importance of developing regionally tailored policies and programs, as a “one-size-fits-all” approach is unlikely to be effective. A multifaceted strategy is necessary to address the significant regional disparities and the influence of socioeconomic factors on adolescents fertility rates across Brazilian municipalities. Future research should focus on identifying specific contextual factors that contribute to high rates in particular regions. Such evidence can guide interventions that are regionally focused and culturally sensitive, aiming to empower girls and their families.

To effectively address adolescent childbearing, interventions must be comprehensive and multisectoral, targeting root causes beyond family planning alone. For instance, qualitative research could explore cultural norms and beliefs influencing adolescent fertility in the North and other deprived regions. By prioritizing tailored interventions, we can work towards reducing adolescent childbearing, thereby saving and improving girls’ lives, enhancing their social contributions, and increasing social capital. Thus, acknowledging and addressing this heterogeneity is not only a matter of improving policy effectiveness but also of promoting social justice and health equity.

## Data Availability

The sources of information used in the study are indicated in the body of the article.
