# Supplementary material for: Maternity in adolescence in Brazil: high fertility rates and stark inequalities across municipalities and regions
Source: Cad Saude Publica. 2026 Feb 6;42:e00057625. doi: 10.1590/0102-311XEN057625 (PMC12884587; doi:10.1590/0102-311XEN057625)
Supplement: Translation

# Translation: Maternidade na adolescência no Brasil: altas taxas de fecundidade e desigualdades gritantes entre municípios e regiões

**DOI:** 10.1590/0102-311XPT057625

**Authors:** Aluísio J. D. Barros, Cauane Blumenberg, Janaína Calu Costa, Luis Paulo Vidaletti Ruas, Natália P. Lima, Fernando César Wehrmeister, Francine dos Santos Costa, Luiza Eunice Sá da Silva, Cesar G. Victora

**Affiliations:** 1 Centro Internacional de Equidade em Saúde, Universidade Federal de Pelotas, Pelotas, Brasil.; 2 Faculdade de Saúde Pública, Universidade de São Paulo, São Paulo, Brasil.

**Keywords:** Nascido Vivo, Mães Adolescentes, Taxa de Fecundidade, Desigualdade em Saúde

## Abstract

A gravidez na adolescência é um grande desafio para a saúde pública no Brasil, com uma taxa de fecundidade de 43,6 nascimentos por mil meninas de 15 a 19 anos em 2022. Este estudo investigou as desigualdades nas taxas de fecundidade adolescente entre municípios e regiões brasileiras e explorou a associação entre taxas de fecundidade adolescente e características sociodemográficas, especificamente, privação municipal e tamanho populacional. Foram utilizados dados do SINASC e do *Censo Demográfico* de 2022. As análises incluíram nascimentos entre 2020 e 2022, excluindo municípios com menos de 50 nascimentos no período. As taxas de fecundidade adolescente municipais foram comparadas com a estimativa média observada em países classificados por nível de renda e descritas de acordo com quintis do Índice Brasileiro de Privação e tamanho populacional. As desigualdades dentro de cada região geográfica foram avaliadas utilizando a diferença absoluta média para a média regional e a amplitude. Foram encontradas disparidades substanciais nas taxas de gravidez na adolescência entre as regiões, com o Norte e o Nordeste apresentando as estimativas mais altas, enquanto o Sul e o Sudeste apresentaram taxas comparativamente mais baixas. A Região Centro-oeste apresentou um valor intermediário. Encontramos grandes desigualdades entre os municípios e uma pequena proporção deles, concentrada na Região Norte, apresentou taxas de fecundidade adolescente excepcionalmente alta. Municípios com níveis mais elevados de privação apresentaram taxas de gravidez na adolescência significativamente mais alta, ressaltando a influência de fatores socioeconômicos na gravidez entre as adolescentes. Os resultados enfatizam a necessidade de intervenções e políticas direcionadas que abordem esses determinantes contextuais para reduzir efetivamente as taxas de gravidez entre meninas no Brasil.

## Introdução

A gravidez na adolescência continua sendo um grande desafio de saúde pública no Brasil. De acordo com estimativas do Banco Mundial (conjunto de dados v2_898), em 2022, a taxa de fecundidade adolescente do Brasil foi estimada em 43,6 nascimentos por mil meninas de 15-19 anos por ano, superando seus pares do BRICS, ou seja, Rússia, Índia e China, com uma taxa de fecundidade adolescente máxima de 14,2 ^1^. A taxa do Brasil também é maior do que a de seus pares da categoria de renda média-alta, cuja média é de 27,8. Embora os níveis ainda sejam muito altos, o país tem mostrado um declínio na fecundidade e maternidade entre as mulheres jovens nos últimos anos; no entanto, esse declínio tem sido desigual entre os grupos sociodemográficos ^2^ ^,^ ^3^ ^,^ ^4^. 

A alta taxa de fecundidade adolescente no Brasil contrasta com a baixa taxa de fecundidade total, 1,6 em 2023, abaixo da taxa de fecundidade de reposição, e o alto acesso à contracepção, revelado pela grande proporção de mulheres em idade reprodutiva que usam contraceptivos modernos (84% em 2019) ^5^ ^,^ ^6^. Isso sugere que os programas de planejamento familiar não estão alcançando adequadamente as adolescentes, ou que aquelas que vivem em contextos desfavoráveis com limitações nas oportunidades de vida, no acesso à educação ou nos serviços de saúde têm maior probabilidade de ter uma gravidez precoce ^7^. 

Ter um filho na adolescência implica vários resultados adversos que afetam a saúde, a educação e a estabilidade econômica. Os riscos à saúde incluem maiores ocorrências de complicações, como anemia, trabalho de parto prematuro e bebês com baixo peso ao nascer, devido à imaturidade fisiológica das mães jovens ^8^ ^,^ ^9^ ^,^ ^10^. Educacionalmente, as mães adolescentes muitas vezes enfrentam contratempos significativos, incluindo taxas de evasão mais altas e desempenho acadêmico limitado, o que, por sua vez, restringe oportunidades futuras ^11^ ^,^ ^12^ ^,^ ^13^ ^,^ ^14^. Economicamente, as mães jovens são mais propensas a experimentar instabilidade financeira e pobreza, pois enfrentam desafios para garantir um emprego estável devido à interrupção da educação e às suas responsabilidades parentais ^15^ ^,^ ^16^. Assim, a gravidez na adolescência é um resultado indesejável para a menina, sua família e sociedade, resultando em um menor nível de desenvolvimento social. Além disso, é essencial notar que os riscos associados à gravidez para adolescentes mais jovens (menos de 14) e mais velhas (15-19) são bastante diferentes, sendo que o primeiro grupo, de acordo com a legislação brasileira, se caracteriza como estupro de vulnerável (artigo 217-A do *Código Penal Brasileiro* ^17^). Portanto, apesar do menor número, as meninas menores de 14 anos não devem ser ignoradas ao estudar a gravidez na adolescência. 

Embora vários fatores de nível individual possam influenciar a fecundidade adolescente, como o desempenho educacional ^18^ e um efeito intergeracional ^19^, as desigualdades subnacionais e as características comunitárias e contextuais têm sido destacadas como alguns dos vários aspectos que afetam sua ocorrência ^7^ ^,^ ^20^ ^,^ ^21^.

Portanto, identificar quem são as mães adolescentes, onde moram e o que impulsiona esse fenômeno é essencial para entender o processo e traçar políticas para diminuir as taxas de fecundidade adolescente no país. 

Este estudo investigou as desigualdades na taxa de fecundidade adolescente entre os municípios brasileiros utilizando dados do Sistema de Informações sobre Nascidos Vivos (SINASC). O estudo teve como objetivo expor as disparidades dessas taxas entre municípios e regiões, identificar áreas com maior ocorrência e avaliar a associação entre vulnerabilidade social e maternidade precoce.

## Métodos

Este estudo utilizou dados do SINASC ^22^ de 2020 a 2022, obtidos através do OpenDataSUS ^23^. Além disso, as estatísticas populacionais por sexo e idade do *Censo Demográfico* de 2022 foram obtidas do Instituto Brasileiro de Geografia e Estatística (IBGE) ^24^.

As taxas de fecundidade adolescente foram calculadas dividindo-se o número de nascimentos entre adolescentes de 10-14 anos e de 15-19 anos ocorridos entre 2020 e 2022 em cada município por três vezes a sua população estimada a partir do *Censo Demográfico* de 2022, de forma a anualizar a taxa. Foram excluídos da análise os municípios com menos de 50 nascimentos nos três anos. Os dados relativos a três anos foram utilizados para garantir melhor estabilidade das estimativas e reduzir possíveis ruídos, considerando que os nascimentos de meninas com menos de 20 anos são apenas cerca de 13% do total. Ao estimar a mediana e a média da taxa de fecundidade adolescente por grupos de municípios, as taxas foram ponderadas pelo tamanho da população correspondente.

Em vista da falta de uma classificação para níveis de taxa de fecundidade adolescente, criamos um critério de comparação a partir das estimativas médias de taxa de fecundidade adolescente observadas em grupos de países classificados por nível de renda do Banco Mundial, com base nos valores de 2022: alta renda (11,2 nascimentos por mil meninas adolescentes), renda média-alta (27,8), renda média-baixa (44,7) e renda baixa (94,0) (API_SP. ADO.TFRT_DS2_en_excel_v2_898.xlsx) ^1^. Os pontos médios aproximados entre esses valores foram usados como pontos de corte para criar faixas de taxa de fertilidade: < 20, 20 |-- 40, 40 |-- 65, 65+ para países de renda alta, média-alta, média-baixa e baixa, respectivamente. A partir disso, foram calculados o número e a proporção de municípios brasileiros com taxa de fecundidade adolescente situada dentro de cada faixa.

A desigualdade municipal dentro das regiões geográficas (Norte, Nordeste, Sudeste, Sul e Centro-oeste) foi avaliada por meio de dois indicadores: a diferença absoluta média para a média regional (DAMM) e a amplitude. A DAMM é definida como $\frac{\sum \left|y_{i}-\bar{y}\right|}{N}$ , onde *y*_*i*_ é a taxa de fecundidade adolescente municipal, ӯ é a média regional e *N* é o número de municípios dentro de cada região. A amplitude foi calculada como a taxa máxima menos a mínima dentro das regiões.

O Índice Brasileiro de Privação (IBP) ^25^ foi utilizado para avaliar o nível de privação municipal. Esse indicador multidimensional combina dados sobre (i) o percentual de domicílios com renda inferior a meio salário mínimo, (ii) o percentual de analfabetos de sete anos ou mais de idade e (iii) o percentual da população sem acesso adequado a esgoto, água potável e coleta de lixo, e sem banheiro/chuveiro. O índice não inclui mortalidade infantil ou expectativa de vida, como o Índice de Desenvolvimento Humano (IDH), o qual pode ser afetado pelo nível de taxa de fecundidade adolescente. A última versão disponível desse índice é baseada em dados do *Censo Demográfico* de 2010. Os municípios foram classificados pelo IBP e divididos em cinco grupos de igual tamanho, não ponderados pela população, para garantir um número igual de unidades em cada grupo, em vez de uma contagem populacional igual. Além disso, o porte populacional (categorizado em < 5 mil, 5 mil, 10 mil, 50 mil, 100 mil+ pessoas) foi explorado como um potencial preditor de taxa de fecundidade adolescente, fornecendo contexto adicional aos padrões de fecundidade observados.

A estimação da taxa de fecundidade adolescente por quintis de IBP e tamanho do município por região geográfica empregou modelos de regressão linear, incluindo uma interação entre os preditores e a região, com termos de interação testados quanto à significância. Todas as análises estatísticas e gráficos foram feitos com o Stata (https://www.stata.com). 

As análises utilizaram dados anônimos de fontes disponíveis publicamente, que não requerem autorização ética.

## Resultados

Um total de 7.968.916 registros de nascimento de 2020 a 2022 foram recuperados do SINASC. Destes, 49.325 (0,62%) nascimentos foram de meninas de 10-14 anos e 1.012.640 (12,7%) de adolescentes de 15-19 anos. A informação sobre a idade estava faltando em apenas 141 registros. Após a exclusão de 68 municípios com menos de 50 nascimentos de adolescentes (10-19 anos), a amostra analítica final foi composta por 5.502 municípios (98,8% dos municípios brasileiros).

A mediana municipal de taxa de fecundidade adolescente para meninas de 10-14 anos foi de 1,9 nascimentos por mil, enquanto a mediana para aquelas de 15-19 anos foi substancialmente maior, de 43,3 nascimentos por mil, variando de zero a 201. A distribuição da taxa de fecundidade adolescente para a faixa de 15-19 anos se assemelha a uma distribuição normal com desvio para a direita (Figura 1), indicando uma proporção pequena, mas um número relevante de municípios com taxas de fecundidade adolescente muito altas. A Figura 1 também indica que 22% dos municípios se enquadraram na faixa de taxa de fecundidade adolescente de países de baixa renda (estimativas superiores a 65 nascimentos por mil) e 47% na faixa de renda média-baixa. Apenas 28% se enquadraram na faixa de renda média-alta, à qual pertence o Brasil, e 3% na faixa de renda alta.

**Figura 1:** Distribuição da taxa de fecundidade na adolescência (15-19 anos) em 5.502 municípios brasileiros, 2020-2022.

Nota: o histograma é dividido em bandas de acordo com os valores médios de taxa de fecundidade adolescente em países de renda alta, média-alta, média-baixa e baixa. Na legenda, ao lado dos rótulos das bandas, indicamos a porcentagem de municípios que se enquadram em cada faixa.

Disparidades regionais substanciais nas taxas de fecundidade adolescente ficaram evidentes. Para jovens adolescentes de 10-14 anos, a Região Norte exibiu uma taxa de fecundidade adolescente mediana quase quatro vezes maior do que a Sul. As estimativas medianas da taxa de fecundidade adolescente da região (em nascimentos por mil) foram, em ordem decrescente, 4,0 (Norte), 2,8 (Nordeste), 2,0 (Centro-oeste), 1,2 (Sudeste) e 1,1 (Sul). Dez municípios do país apresentaram taxas de fecundidade adolescente de 20 ou mais para as meninas de 10-14 anos (Figura 2a).

**Figura 2:** Taxas de fecundidade na adolescência para 5.502 municípios brasileiros por região geográfica e a mediana para cada região, 2020-2022.

Nota: o gráfico 2a) mostra as taxas para adolescentes de 10-14 anos, e o 2b) para adolescentes de 15-19 anos. O gráfico 2b) também mostra os percentuais de municípios em cada faixa de taxa de fecundidade definida pelas taxas médias de países de alta, média-alta, média-baixa e baixa renda.

Entre as adolescentes de 15-19 anos, a mediana da taxa de fecundidade adolescente da Região Norte (77,1) foi mais que o dobro da do Sul (35,0) (Tabela 1 e Figura 2b). Dentro de cada uma das cinco regiões, as taxas de fecundidade adolescente no nível municipal variaram amplamente e, em todas elas, foram encontrados municípios com níveis baixos e altos de taxa de fecundidade adolescente. Um relevante conjunto de 148 municípios apresentou taxa de fecundidade adolescente de 100 ou mais para meninas de 15-19 anos. 

**Tabela 1:** Mediana da taxa de fecundidade adolescente (15-19 anos) por região geográfica e em nível nacional, número e proporção de municípios com taxas de fecundidade na adolescência dentro de faixas definidas pela taxa média de países de alta, média-alta, média-baixa e baixa renda. Brasil, 2020-2022.

Região geográfica	Todos os municípios		Faixas da taxa de fecundidade adolescente (nascimentos/1.000)			
	N	Mediana (taxa de fecundidade adolescente/1.000)	< 20 faixa renda alta	20 |-- 40 faixa renda média-alta	40 |-- 65 faixa renda média-baixa	65+ faixa renda baixa
			% municípios	% municípios	% municípios	% municípios
Norte	450	77,1	0,2	1,8	22,0	76,0
Nordeste	1.793	52,8	0,2	11,6	57,7	30,5
Sudeste	1.638	35,8	2,9	46,7	45,3	5,1
Sul	1.162	35,0	8,8	41,8	40,0	9,4
Centro-oeste	459	46,6	1,1	11,3	54,9	32,7
Total	5.502	43,3	2,9	27,6	47,1	22,4

Nota: a tabela inclui 5.502 municípios com 50 ou mais nascimentos de adolescentes de 10-19 anos de 2020 a 2022.

A Figura 2b também apresenta o percentual de municípios na faixa de taxa de fecundidade adolescente de cada grupo de renda para adolescentes de 15-19 anos, por região. A Região Norte apresentou o maior percentual de municípios (76%) enquadrados na faixa de países de baixa renda, contrastando com as regiões Sudeste e Sul, que apresentaram distribuição mais favorável, embora ainda com metade dos municípios dentro das faixas de renda média-baixa e baixa. Nenhuma das regiões apresentou a maioria dos municípios dentro da faixa de renda média-alta do Brasil.

As medidas das desigualdades municipais dentro das regiões, visualizadas nos gráficos, são apresentadas na Tabela 2. Para as meninas de 10-14 anos, as regiões Norte e Centro-oeste apresentaram as maiores desigualdades, com valores de DAMM marcadamente mais altos em comparação com as demais regiões. Para os adolescentes de 15-19 anos, as regiões Norte e Centro-oeste também apresentaram os maiores valores de DAMM, mas não na mesma proporção que as regiões Nordeste e Sul. As medidas confirmam a impressão visual de altas desigualdades em todas as cinco regiões. As amplitudes foram superiores a 100 nascimentos por mil em três regiões, indicando enormes desigualdades. 

**Tabela 2:** Diferença absoluta média em relação à média regional (DAMM) e amplitude para as taxas de fecundidade na adolescência por região geográfica. Brasil, 2020-2022.

Região geográfica	Idade 10-14 anos		Idade 15-19 anos	
	DAMM	Amplitude	DAMM	Amplitude
Norte	3,1	28,9	20,5	117,6
Nordeste	1,7	17,1	13,0	73,8
Sudeste	1,3	22,7	10,3	63,6
Sul	1,6	13,5	13,4	114,0
Centro-oeste	2,3	37,8	14,5	107,5

Nota: amplitude − diferença entre os valores máximo e mínimo dentro da região.

A taxa de fecundidade adolescente municipal se mostrou fortemente associada ao IBP (Figura 3 e Tabelas 3 e 4). O modelo de regressão, incluindo o IBP, a região geográfica e sua interação, explicou 64,6% da variabilidade da taxa de fecundidade adolescente na faixa etária de 15-19 anos. Com exceção da Região Norte, os níveis de taxa de fecundidade adolescente para o mesmo quintil de privação em cada região não foram muito diferentes. Além disso, foi observado um aumento semelhante na taxa de fecundidade adolescente com a privação para todas as regiões. Na Região Norte, encontramos taxas de fecundidade adolescente sistematicamente maiores em cada quintil do IBP em comparação com outras regiões, exceto para o grupo menos carente, que possuía apenas um município. Note que na Região Sul nenhum município estava no grupo de maior privação. A Região Centro-oeste apresentou uma taxa de fecundidade adolescente muito maior para o grupo mais carente; no entanto, essa estimativa é baseada em apenas três municípios.

**Figura 3:** Taxas médias de fecundidade na adolescência (15-19 anos) para os municípios em cada quintil do Índice Brasileiro de Privação (IBP) por região geográfica. Brasil, 2020-2022.

Nota: os números em algumas barras indicam o número de municípios em cada grupo quando menos de 10. Quintis de privação: 1 = menor privação até 5 = maior privação.

**Tabela 3:** Taxas de fecundidade na adolescência (nascimentos por 1.000 meninas adolescentes de 15-19 anos) por região geográfica e quintis do Índice Brasileiro de Privação (IBP).

Região geográfica	IBP (quintis)				
	Q1 (menor privação)	Q2	Q3	Q4	Q5 (maior privação)
Norte	40,9	60,4	72,8	85,0	101,1
Nordeste	36,6	43,5	52,1	58,2	63,5
Sudeste	34,9	46,4	48,0	47,8	48,4
Sul	33,8	45,7	53,8	69,4	0,0
Centro-oeste	39,0	52,3	63,8	75,1	127,8

Nota: estimativas derivadas de um modelo de regressão linear incluindo região geográfica, quintis de IBP e sua interação (todos os valores de p < 0,001). O R^2^ para o modelo foi de 0,646.

**Tabela 4:** Número de municípios em cada quintil e região geográfica do Índice Brasileiro de Privação (IBP).

Região geográfica	IBP (quintis)				
	Q1 (menor privação)	Q2	Q3	Q4	Q5 (maior privação)
Norte	1	19	104	193	132
Nordeste	4	21	127	686	955
Sudeste	646	491	312	167	22
Sul	411	416	305	27	0
Centro-oeste	26	136	253	40	3

Nota: cinco municípios não possuíam estimativa de IBP.

O porte do município apresentou associação estatisticamente significativa com a taxa de fecundidade adolescente para meninas de 15-19 anos, mas com magnitude bem menor do que a de privação. De modo geral, observou-se associação em formato de U invertido com a taxa de fecundidade adolescente máxima em municípios com população entre 10 e 50 mil pessoas. Esses resultados são apresentados na Figura 4 e nas Tabelas 5 e 6. Adicionamos o tamanho da população a um modelo incluindo o IBP para avaliar o poder explicativo adicional do tamanho municipal ao IBP. O tamanho da população permaneceu significativo quando ajustado para privação, mas o coeficiente de determinação do modelo (R^2^) aumentou apenas 0,4 pontos percentuais (dados não apresentados).

**Figura 4:** Taxas médias de fecundidade na adolescência (15-19 anos) para municípios em cada grupo de porte populacional por região geográfica. Brasil, 2020-2022.

**Tabela 5:** Taxas de fecundidade na adolescência (nascimentos por 1.000 meninas adolescentes de 15-19 anos) por região geográfica e tamanho do município.

Região geográfica	Porte do município (população total)				
	< 5.000	5.000	10.000	50.000	100.000
Norte	77,1	81,1	93,4	82,4	64,4
Nordeste	57,5	59,2	60,6	58,3	47,0
Sudeste	42,9	42,7	42,9	38,9	35,6
Sul	40,9	43,6	43,9	39,4	32,4
Centro-oeste	56,8	62,4	62,3	56,8	40,7

Nota: estimativas derivadas de um modelo de regressão linear incluindo região geográfica, grupos de tamanho do município e sua interação (todos os valores de p < 0,001). O R^2^ para o modelo foi de 0,564.

**Tabela 6:** Número de municípios em cada grupo de porte populacional e região geográfica.

Região geográfica	Porte do município (população total)				
	< 5.000	5.000	10.000	50.000	100.000
Norte	92	66	221	45	26
Nordeste	247	372	999	111	64
Sudeste	367	374	640	108	149
Sul	412	262	378	55	55
Centro-oeste	138	96	181	19	25

## Discussão

Este estudo revela desigualdades significativas nas taxas de fecundidade na adolescência em mais de cinco mil municípios brasileiros no período de 2020 a 2022. Houve desigualdades marcantes na taxa de fecundidade adolescente entre os municípios do país, bem como dentro das regiões geográficas - as quais possuem populações mais homogêneas.

A comparação da taxa de fecundidade adolescente em adolescentes de 15-19 anos com países de diferentes níveis de renda revelou que 70% dos municípios brasileiros apresentaram taxas superiores às dos países de renda média-alta, nível do próprio Brasil. A situação é crítica na Região Norte, onde 98% dos municípios estão acima desta faixa. Também mostramos que uma grande proporção da variabilidade municipal nas taxas de fecundidade adolescente é explicada pelo nível de privação municipal, o que oferece uma pista importante sobre o que pode ser feito para reduzir as altas taxas observadas.

A substancial variabilidade regional e municipal observada nas taxas de fecundidade adolescente, particularmente entre meninas de 15-19 anos, revela um cenário complexo e desigual da maternidade na adolescência no Brasil. O forte efeito da privação municipal sobre as taxas de fecundidade adolescente ressalta o papel crucial dos fatores socioeconômicos e do desenvolvimento humano na maternidade na adolescência, bem como a influência estrutural da desigualdade social na formação desse resultado. Outros estudos relataram a associação entre taxa de fecundidade adolescente e pobreza, falta de acesso aos serviços de saúde ^26^ e baixa escolaridade ^27^. Nossos achados estão alinhados com a literatura anterior que descreveu a distribuição nacional heterogênea de nascimentos entre adolescentes em todo o Brasil e a associação entre esses nascimentos e medidas contextuais, como o IDH Municipal e a desigualdade de renda, medida pelo coeficiente de Gini ^20^ ^,^ ^28^. Combater a pobreza, melhorar o acesso à educação - especialmente para meninas - e melhorar os serviços de planejamento familiar são aspectos primordiais para intervenções eficazes. Além disso, a falta de perspectivas de longo prazo para uma melhor qualidade de vida pode prejudicar a capacidade dos adolescentes de planejar com antecedência e levá-los a soluções mais imediatas, como ter filhos em busca de maior status social, segurança e companhia ^29^.

As taxas de fecundidade adolescente consistentemente mais altas na Região Norte, em todos os níveis de privação, destacam a complexa interação de fatores socioeconômicos e outras influências potenciais específicas da região. Esses fatores podem incluir normas culturais, acessibilidade aos serviços de saúde e disponibilidade de recursos de planejamento familiar ^28^. Fatores como maior proporção de populações indígenas, distância, acesso limitado a serviços de saúde e níveis mais altos de privação justificam uma investigação mais aprofundada. As políticas públicas nessas áreas devem integrar ações intersetoriais, incluindo educação sexual e reprodutiva integral nas escolas, ampliação do acesso a métodos contraceptivos e fortalecimento da atenção primária à saúde com foco na saúde do adolescente e da mulher. A presença de municípios com alta taxa de fecundidade adolescente mesmo em regiões com melhores indicadores gerais, como Sudeste e Sul, destaca a importância de identificar focos de vulnerabilidade em ambientes aparentemente mais favorecidos. Essa heterogeneidade sugere que intervenções uniformes em todo o país podem ser insuficientes e reforça a necessidade de estratégias sob medida, adaptadas a contextos regionais e locais específicos. 

O número de nascimentos de adolescentes de 10-14 anos é particularmente preocupante, apesar de ser uma pequena proporção do total. Encontramos 49.325 nascimentos de meninas nessa faixa etária em 2020-2022, uma média de 16.441 nascimentos por ano. De acordo com a legislação brasileira, gestações de meninas menores de 14 anos são consideradas resultado de estupro de vulnerável, o que exige ações imediatas e rigorosas para eliminar tais nascimentos com iniciativas de proteção às meninas e garantia de seus direitos sexuais e reprodutivos. Nos casos em que a sociedade não consegue evitar uma gravidez nessa faixa etária, o acesso fácil e rápido ao aborto legal, quando esta for sua escolha, deve ser garantido.

Este estudo se beneficia da alta cobertura de dados do SINASC, parto institucional quase universal em todos os grupos sociais e municípios, baixíssima falta de informação, abrangência nacional e grande tamanho amostral. No entanto, como um estudo ecológico baseado em dados secundários, a pesquisa tem capacidade limitada de explorar fatores de nível individual que influenciam a fecundidade na adolescência. Pesquisas adicionais integrando abordagens de métodos mistos podem aumentar a compreensão dessas relações complexas. Nossas análises se concentraram em nascidos vivos, não incluindo, portanto, natimortos e abortos. Consequentemente, não apresentamos um quadro completo da gravidez na adolescência, embora a maioria dos eventos esteja certamente representada aqui. O número relativamente pequeno de municípios nos quintis mais carentes em certas regiões limita a generalização dos achados para esses contextos. Observe que usamos um IBP desenvolvido com os dados do Censo de 2010, uma vez que uma versão atualizada com dados do Censo de 2022 ainda não está disponível. Portanto, as mudanças na privação municipal desde 2010 não são capturadas em nossa análise. Mesmo assim, a associação encontrada foi clara e forte. 

Também é importante considerar que o período do estudo (2020-2022) coincide com a pandemia de COVID-19. Esse contexto pode ter influenciado os padrões de fecundidade na adolescência em todo o Brasil, pois prejudicou o acesso aos serviços de saúde, incluindo a oferta de anticoncepcionais e a atenção à saúde sexual e reprodutiva, particularmente em regiões já com fragilidades estruturais. O fechamento de escolas e a redução do acesso à educação e aos programas de proteção social podem ter aumentado ainda mais as vulnerabilidades entre os adolescentes, especialmente em áreas carentes. Embora nossos dados não possam isolar os efeitos específicos da pandemia, as disparidades observadas na taxa de fecundidade adolescente podem refletir parcialmente o impacto desigual do COVID-19 entre regiões e municípios, potencialmente exacerbando as desigualdades pré-existentes. Esse contexto reforça a importância de sistemas sociais e de saúde resilientes e equitativos, capazes de manter serviços essenciais durante as crises. Além disso, trabalhos anteriores revelaram que os efeitos das crises, incluindo a pandemia de COVID-19, foram menores do que inicialmente especulado, dada a tendência de queda pré-existente ^4^.

Os resultados destacam a importância de desenvolver políticas e programas adaptados para realidades regionais e locais, pois é improvável que uma abordagem de “tamanho único” seja eficaz. Uma estratégia multifacetada é necessária para abordar as disparidades regionais significativas e a influência de fatores socioeconômicos nas taxas de fecundidade na adolescência nos municípios brasileiros. Pesquisas futuras devem se concentrar na identificação de fatores contextuais que contribuem para altas taxas em regiões específicas. Esta pesquisa pode orientar intervenções com foco regional e culturalmente sensíveis, com o objetivo de empoderar as meninas e suas famílias. 

Para combater efetivamente a gravidez na adolescência, as intervenções devem ser abrangentes e multissetoriais, abordando as causas profundas além do planejamento familiar. Por exemplo, a pesquisa qualitativa pode explorar normas e crenças culturais que influenciam a fertilidade adolescente na Região Norte e em outras áreas carentes. Ao priorizar essas intervenções personalizadas, podemos trabalhar para reduzir a gravidez na adolescência, salvando e melhorando a vida das meninas, aumentando suas contribuições sociais e aumentando o capital social. Assim, reconhecer e abordar essa heterogeneidade não é apenas uma questão de melhorar a eficácia das políticas, mas também de promover a justiça social e a equidade na saúde.

## Data Availability

As fontes de informação utilizadas no estudo estão indicadas no corpo do artigo.
